# Profiling of Homocysteine Metabolic Pathway Related Metabolites in Plasma of Diabetic Mellitus Based on LC-QTOF-MS

**DOI:** 10.3390/molecules28020656

**Published:** 2023-01-09

**Authors:** Chanyi Li, Jiaying Qin, Wuping Liu, Bo Lv, Ning Yi, Jinfeng Xue, Zhigang Xue

**Affiliations:** 1 Department of Regenerative Medicine, School of Medicine, Tongji University, 1239 Siping Road, Shanghai 200092, China; 2 International Joint Research Center for Medical Metabolomics, Xiangya Hospital, Central South University, 87 Xiangya Road, Changsha 410008, China; 3 Translational Center of Stem Cell Research, Tongji Hospital, Tongji University School of Medicine, Shanghai 200065, China; 4 Hunan Jiahui Genetics Hospital, 72 Xiangya Road, Changsha 410008, China

**Keywords:** homocysteine metabolism, diabetes, LC-MS, clinical diagnosis

## Abstract

Background: Homocysteine (Hcy) has been found to be closely related to the occurrence of diabetes mellitus (DM) and is considered as one of the risk factors of DM. However, Hcy alone is not enough as a factor to predict DM, and our study analyzed and determined the relationship between the main metabolites involved in the Hcy metabolic pathway and DM. Methods: A total of 48 clinical samples were collected, including 18 health control samples and 30 DM samples. All standards and samples were detected by LC-QTOF-MS. Multivariate statistical analysis and k-means cluster analysis were performed to screen and confirm the metabolites significantly correlated with DM. Results: A total of 13 metabolites of the Hcy metabolic pathway were detected in the samples. The content of Hcy, cysteine, taurine, pyridoxamine, methionine, and choline were significantly increased in the DM group (*p* < 0.05). Hcy, choline, cystathionine, methionine, and taurine contributed significantly to the probabilistic principal component analysis (PPCA) model. The odds ratios (OR) of Hcy, cysteine, taurine, methionine, and choline were all greater than one. K-means cluster analysis showed that the Hcy, taurine, methionine, and choline were significantly correlated with the distribution of glucose values (divided into four levels: 10.5–11.7 mmol/L, 7.7–9.7 mmol/L, 6.0–6.9 mmol/L, and 5.0–5.9 mmol/L, respectively). Conclusion: Hcy, taurine, methionine, and choline can be used as risk factors for diabetes diagnosis and are expected to be used for the assessment of diabetes severity.

## 1. Introduction

Diabetes mellitus (DM) is one of the most common metabolic diseases and its complications have seriously threatened human health. According to previous reports, 415 million people live with diabetes worldwide, and an estimated 193 million people have undiagnosed diabetes [1]. At present, the diagnosis of DM mainly includes fasting serum/blood glucose and 2-hour blood glucose value in oral glucose test (OGTT), which requires patients to fast for at least 8 h. HbA1c is glucosylated hemoglobin, which is the combination of hemoglobin in red blood cells and blood glucose over a long time. HbA1c has become the gold standard for monitoring blood glucose control in diabetic patients, but different race, age and clinical factors still raise doubts about the results of using a single HbA1c [2]. DM is a huge and increasingly serious clinical and public health problem, which causes concern in global medical staff, and its financial burden is huge and growing. Therefore, the improvement of early diagnosis technology plays an important role in early intervention [3,4].

Homocysteine (Hcy) is an important intermediate sulfur-containing amino acid in the metabolism of methionine and cysteine. A large number of studies have shown that Hcy is an important risk factor for systemic atherosclerosis and cardiovascular disease [5,6,7,8], and cardiovascular disease is the largest cause of morbidity and mortality associated with DM [1]. Clinical studies have shown that patients with high blood Hcy have an increased risk of DM complications [9,10]. In addition, studies have shown that certain diseases such as neonatal defects, arteriovenous thrombosis, diabetes, stroke, Alzheimer’s, and Parkinson’s disease are also associated with abnormal Hcy metabolism [9,11,12]. Since the high concentrations of Hcy can induce the risk of various diseases, a variety of methods for the detection of Hcy have been developed clinically to facilitate clinical diagnosis and prevention [13,14,15]. The biomarkers of DM have been studied by metabolomics, which also involve the metabolites on Hcy metabolism [16].

**Figure 1:**
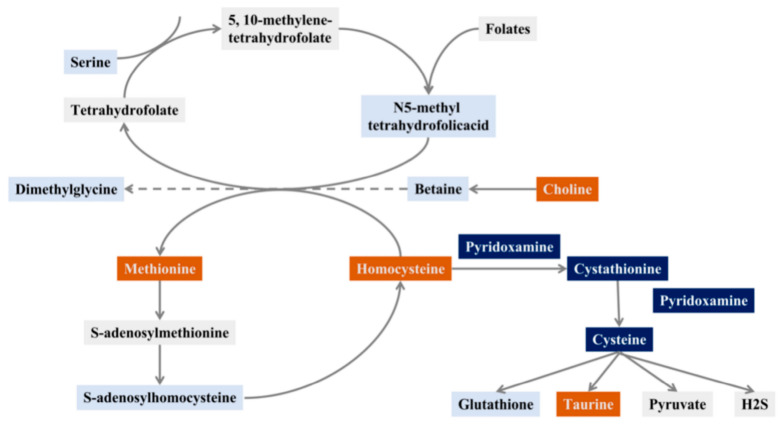
Schematic diagram of the Hcy pathway (orange: screening out metabolites closely related to diabetes, dark blue: screening potential metabolites related to diabetes, light blue: this study detected, but no metabolite related to DM was found, grey: unscreened metabolites in the sample).

Hcy is an important intermediate in folate, vitamin B12, and one carbon metabolism [17]. There are two main metabolic pathways of Hcy: methylation and sulfurization. Transmethylation refers to the conversion of Hcy into methionine, which enters the methionine cycle; this requires 5-methyltetrahydrofolate (5-MTHF) to provide methyl donors. The methionine cycle provides the body with methyl groups for protein synthesis. Sulfurization refers to the synthesis of cystathionine from Hcy, which further forms cystine, and further metabolized to glutathione with antioxidant capacity and the biogaseous mediator hydrogen sulfide (H2S) [18,19] (Figure 1). Other metabolites in the Hcy metabolic pathway are also important for the occurrence and development of DM. Metabolomics studies of the DM population have found that abnormal methionine cycle is closely related to the progression of DM [20]. Therefore, the occurrence and development of DM can be observed more comprehensively by assessing changes in metabolites in the Hcy synthesis and metabolism pathway, as it is often inaccurate to detect only such an indicator as Hcy in clinical practice.

Therefore, we established a DM pseudo-targeted metabolomics study based on liquid chromatography coupled with quadrupole time of flight tandem mass spectrometry (LC-QTOF-MS) technology, and screened out the characteristic Hcy metabolic pathway-related metabolism through multivariate statistical analysis. Through the blood glucose levels of these DM patients, it is verified that these important metabolites are closely related to the grading of blood sugar levels. These data provide new strategies for early diagnosis methods for the occurrence and development of DM.

## 2. Results and Discussion

### 2.1. Determination of Metabolites Related to Homocysteine Metabolic Pathway

**Figure 2:**
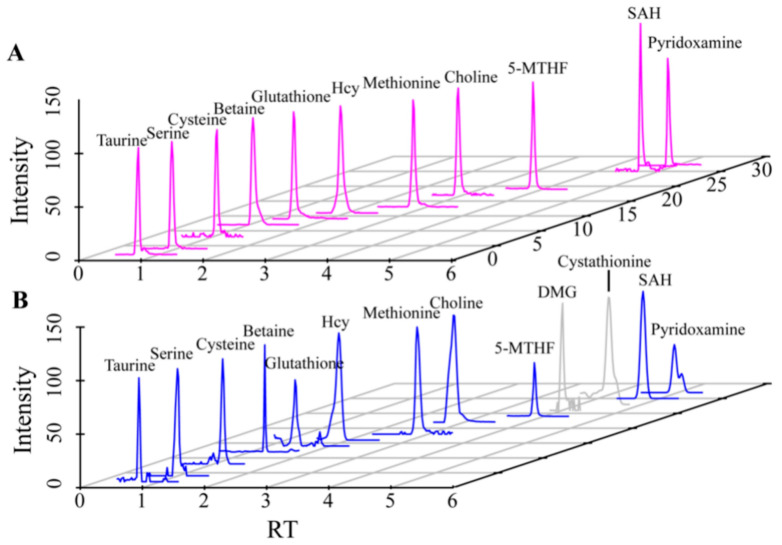
EIC plots of standards and samples detected by LC-QTOF-MS ((**A**). Standards, (**B**). Samples). The metabolites shown from left to right are taurine, serine, cysteine, betaine, glutathione, Hcy, Methionine, choline, 5-MTHF, DMG, cystathionine, SAH, pyridoxamine. The metabolites marked in gray are those determined by the MS/MS information.

The metabonomic of 30 DM samples, 18 healthy control (HC) samples and standard mixtures were detected by LC-QTOF-MS, and the extracted ion chromatograms (EIC) obtained is shown in Figure 2. From the EIC of the standard mixtures (Figure 2A), the retention time and mass-to-charge ratio information for different metabolites were determined. From the EIC of the samples, the MS/MS information aligned with the HMDB database were extracted, and identified two other related metabolites, including dimethylglycine (DMG) and cystathionine (Figure 2B). A total of 13 metabolites which related to the Hcy pathway, including taurine, serine, cysteine, betaine, glutathione, Hcy, methionine, choline, 5-MTHF, DMG, cystathionine, SAH, pyridoxamine, were successfully detected. In addition, the information of MS/MS in the HMDB database were also compared to ensure the reliability. The relevant retention times and MS/MS information were shown in Table 1.

### 2.2. Multivariate Statistical Analysis of Metabolites

**Figure 3:**
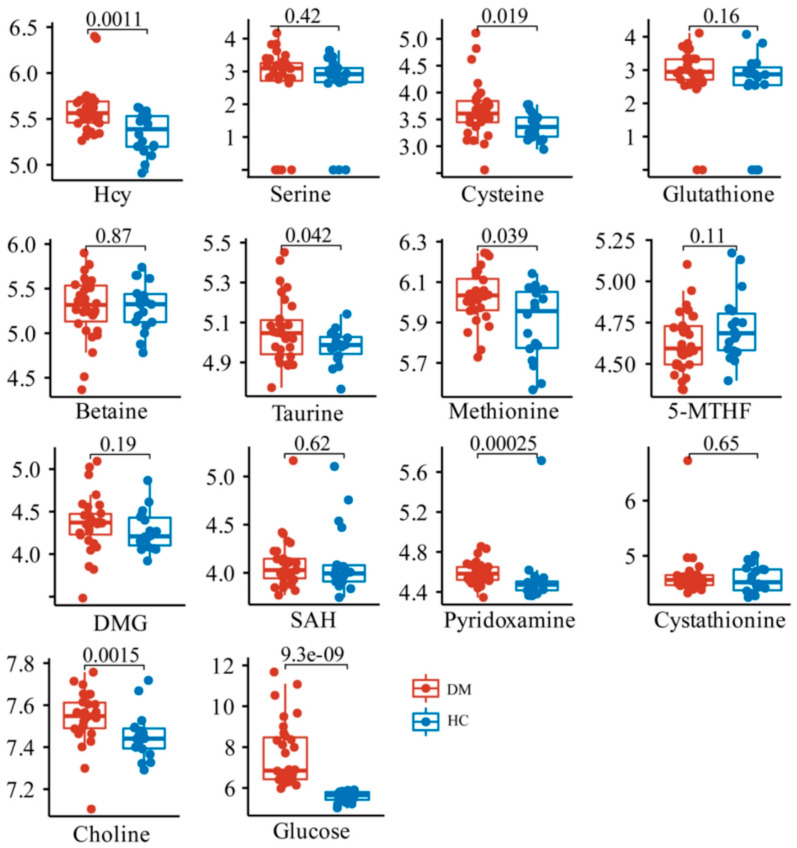
Comparison of relative contents of 13 metabolites in HC and DM groups.

To explore the differences in the relative contents of these metabolites between the HC and DM groups, Kruskal–Wallis and T tests were performed between HC and DM groups. The data showed that the content of Hcy, as well as its downstream metabolites including taurine, cysteine, and pyridoxamine, was significantly increased in the DM group, while the methionine and choline in the folate cycle and methionine cycle were also significantly increased (Figure 3). There were no significant changes in other metabolites related to Hcy synthesis and metabolism.

**Figure 4:**
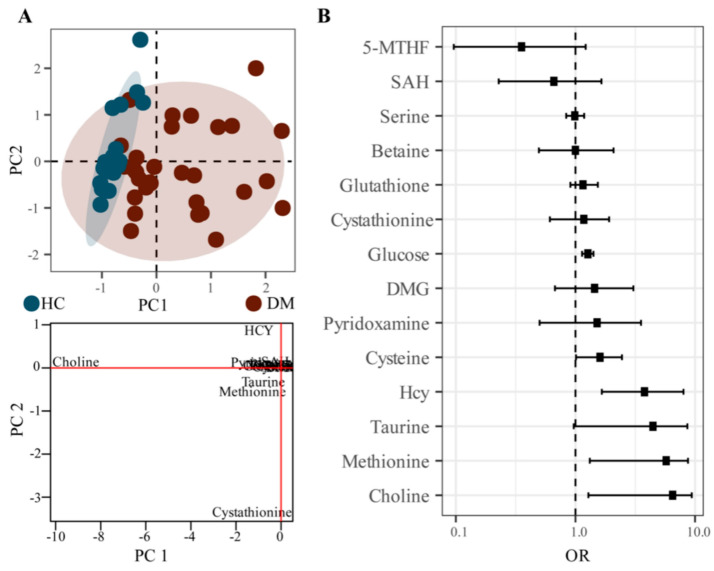
PPCA analysis and Poisson regression analysis of HC and DM groups. ((**A**). PPCA score plot and loading plot, (**B**). OR and confidence interval distribution plot of Poisson regression analysis).

To identify the metabolic characteristics of DM, probabilistic principal component analysis (PPCA) was performed on the metabolite analysis; 13 differential metabolites were identified in the DM group compared with the HC group (Figure 4A). Notably, we found that the Hcy, choline, cystathionine, methionine, and taurine had larger loading values in PC1 or PC2 components and contributed significantly to the PPCA model, suggesting that these metabolites were important variables to distinguish the DM group from the HC group (Figure 4A).

In order to further explore the correlation between Hcy related metabolites and DM, Poisson regression analysis was performed on the metabolite data. The OR of choline, Hcy, methionine, taurine, and cysteine were all greater than one, and the 95% confidence intervals also were greater than one, indicating a strong correlation with the occurrence of DM.

According to the results of multivariate statistical analysis, different statistical methods show that Hcy, taurine, methionine, and choline are highly related to DM, and other metabolites screened, cysteine, pyridoxamine, and cystathionine, have potential relevance to DM.

### 2.3. Characteristic Metabolite Levels Correlate with Diabetic Blood Glucose

**Figure 5:**
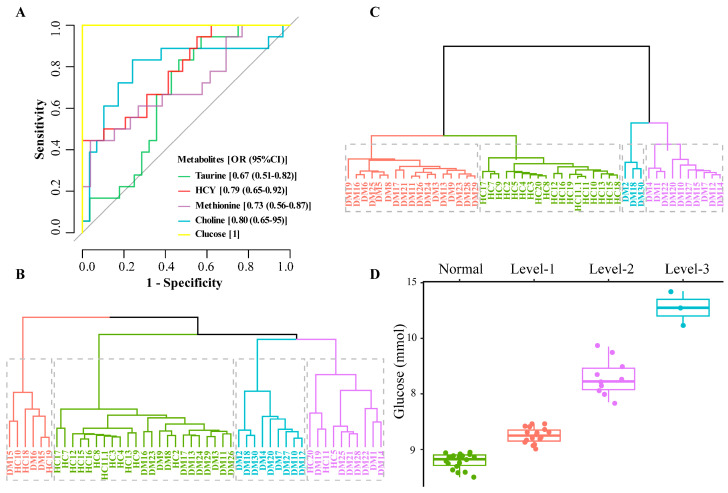
ROC analysis, k-means cluster analysis, and blood glucose distribution corresponding to the clustering results ((**A**). ROC analysis, (**B**). Sample clustering results using all 13 metabolites; 4 clusters (colored and boxed) were identified and HC samples cannot be distinguished from DM samples, (**C**). Sample clustering results based on 4 metabolites; 4 clusters (colored and boxed) were identified and HC samples are in one category, (**D**). Clustering results corresponding to blood glucose distribution).

To further evaluate the potential of these metabolites as early warning information for DM, k-means cluster analysis and receiver operating characteristic (ROC) analysis were performed on the metabolites of Hcy, taurine, methionine, and choline. In ROC analysis, the AUC value of Hcy is 0.79 (0.65–0.92), 0.80 (0.65–0.95) for choline, 0.73 (0.56–0.87) for methionine, and 0.67 (0.51–0.82) for taurine (Figure 5A). The larger the AUC value of the metabolites, the greater the potential as early warning information of DM.

Comparing the k-means cluster analysis of these 13 metabolites which cannot classify HC and DM groups well (Figure 5B), the four biomarkers (Hcy, taurine, methionine, and choline) screened can cluster the HC group into one category (Figure 5C). The cluster analysis further verified that the four metabolites screened out in the multivariate statistical analysis were significantly related to DM, which may be related to the distribution of glucose levels. Blood glucose is an important indicator for the diagnosis of DM, and our multivariate statistical analysis also found that glucose was significantly different between HC and DM groups (Figure 3 and Figure 4B). Further analysis of the clustering results showed that the k-means targets of the DM clustering of the four metabolites were significantly correlated with the distribution of glucose values (Figure 5D). The glucose level of three samples (DM group) in the blue cluster is between 10.5 and 11.7 mmol/L, the glucose concentration of 18 samples (DM group) in the purple cluster is 7.7–9.7 mmol/L, for 17 samples (DM group) in the red cluster it is 6.0–6.9 mmol/L, and all samples in HC group in the green cluster have 5.0–5.9 mmol/L. Therefore, it can be seen that the four metabolites screened out may have a significant role in the DM classification.

The results of our screening of metabolites related to the Hcy metabolic pathway illustrated that Hcy was significantly associated with DM. Methionine involved in the methionine cycle, choline participated in the folate cycle, and taurine, which had a hand in the transsulfurization pathway (Figure 1), were distributed upstream or downstream of Hcy and were closely related to the synthesis and metabolism of Hcy.

The metabolites involved in Hcy metabolism pathways were detected, but there were still some metabolites with extremely low content not detected in this study (Figure 1, gray mark). For example, folate, an important metabolite in the folate cycle, was also not detected in other studies [21]. To detect folate may require specific enrichment in samples, but it was not realistic in actual clinical rapid detection, so this metabolite was not considered in this study for the time being. Other undetected metabolites may be related to the low content in the sample and the applicability of the method.

The four potential risk factors related to DM, including Hcy, taurine, methionine, and choline, that we screened were significantly increased in the DM group. Elevated plasma Hcy levels had been identified as a risk factor for many diseases, including cardiovascular disease. The increase of Hcy is related to DM, possibly through the increase of redox stress and the damage of folate-mediated one-carbon metabolism [22]. The increase of Hcy is a biomarker of diabetic neuropathy, retinopathy, nephropathy and other microvascular complications. In poorly controlled type 2 diabetes, elevated Hcy levels are associated with an increased risk of macrovascular complications such as atherosclerosis and cardiovascular disease [23]. Hcy is also an independent risk factor for DM with heart failure [24]. Cysteine is oxidized and further metabolized to taurine or sulfate, usually metabolized through the desulfurization pathway, producing hydrogen sulfide (H2S). Taurine affects cell function and regulates different cell processes, including osmotic regulation, antioxidation, ionic exercise regulation and bile acid binding, and regulates energy metabolism, gene expression, osmotic, and protein quality control, which has anti-inflammatory effects and can improve DM [25,26]. Methionine is primarily metabolized in the liver, where methionine adenosyltransferase catalyzes methionine to S-adenosylmethionine (SAM), which is a methyl donor for DNA methylation, which is implicated in glucose metabolism, insulin resistance, and beta cell dysfunction in DM [27]. The increased concentration of methionine cycle intermediates in plasma is associated with renal dysfunction in patients with DM [28]. Choline is considered as the central methyl donor required for mitochondrial protein and nucleic acid synthesis via its active forms 5-MTHF and betaine, respectively [29]. Choline has also been found to be associated with T2D with heart failure [24].

In addition, the potential metabolites which can be concerned in the subsequent expansion of sample detection, including cysteine, pyridoxamine, and cystathionine, have also been studied. Cysteine synthesis can be seen as part of the degradation of Hcy, generated through the transsulfuration pathway [30]. Research found that a high level of Hcy can be spontaneously bonded to the cysteine site of the insulin receptor precursor protein through a disulfide bond. This post-translational modification will disrupt the maturation process of the insulin receptor in the endoplasmic reticulum and Golgi apparatus, and ultimately lead to significant reduction of the mature insulin receptor protein [31]. Pyridoxamine is a form of vitamin B6, involved in glutathione biosynthesis from Hcy, and has been proven to reduce the formation of advanced glycosylation end products in DM [32]. DM with incipient nephropathy was associated with pronounced alterations in vitamin B6 metabolism [33]. Cyathione is a sulfur-containing amino acid. In patients with cardiovascular disease, the increase of cyclic cystathione is related to oxidative damage and endothelial dysfunction [34]. We found that cystathionine is potentially related to DM, but the specific mechanism needs further study.

Four metabolites screened in this study were involved in the relationship with DM in other studies, especially Hcy related research, and even involved in the mechanism of research. However, there are few in-depth studies on other metabolites, which is also the direction we need to pay attention to in the future.

The occurrence of DM may bring many complications, such as diabetic nephropathy, diabetic retinopathy, diabetic foot, other metabolic disorders, deterioration of brain tissue, and so on. It has been reported that Hcy could be a predictor of the occurrence of DM [35]. Prediabetes (moderately high blood sugar) is a high-risk state for diabetes, and there is an association between prediabetes and an increased risk of numerous complications [36], the level of blood glucose in the body relating to the risk of DM and its complications. In this study, it was found that k-means clustering of six metabolites can distinguish DM and HC groups well, and the DM group can also be subdivided into different levels of blood glucose concentration, which means that the combination metabolites screened also have the potential to diagnose DM complications. Of course, this requires more data accumulation and verification.

The metabolism of the human body itself changes dynamically. This research proposes the combination of the metabolites with significantly related upstream or downstream metabolites into a panel for the overall evaluation of the diagnosis of DM. In the next step of research, more samples and different complications can be included, the fluctuation range of each metabolite detected can be clarified, and an evaluation model can be established for early diagnosis of DM severity and risk of complications.

## 3. Materials and Methods

### 3.1. Chemicals and Reagents

The information for standards and reagents is as follows: methionine and cysteine were purchased from Jiuding Chemical Co., Ltd. (Suzhou, China). S-adenosylhomocysteine (SAH) was purchased from Leyan Reagent (Shanghai, China). Hcy, serine, and pyridoxamine were purchased from Sigma-Aldrich (St. Louis, MO, USA). Betaine, glutathione and taurine were purchased from Acmec Biochemical Co., Ltd. (Shanghai, China). The 5-MTHF was purchased from Yuanye Bio-Technology Co., Ltd. (Shanghai, China), and the purity of standards are all greater than 98%. Perfluoroheptanoic acid (PFHA) was purchased from Energy Chemical (Shanghai, China). Choline was purchased from CNW Technologies (Düsseldorf, Germany), and acetonitrile (Optima LC/MS grade) was purchased from F&H Co., Ltd. Ascorbic acid was purchased from Aladdin Co., Ltd. (Shanghai, China), methanol (mass spectrometry grade) was purchased from Fisher Company, and TCEP was purchased from Yuanye Bio-Technology Co., Ltd. (Shanghai, China). The experimental water was purified by the water purification system (18.2 MΩ, Sartorius, Göttingen, Germany). All the other reagents are at analytical grade.

### 3.2. Sample Preparation

Samples were collected from the donors individually, which conformed to the inclusion criteria. After collection, all samples were collected, processed and tested at the same time. A total of 48 samples were used in this experiment, including 30 samples in the DM group and 18 samples in the HC group. Samples in the DM group were all from patients diagnosed with type 2 diabetes. Blood collected clinically were centrifuged at 13,000 rpm at 4 °C for 15 min, and the supernatant was taken to obtain plasma. Pipeting 100 μL blood plasma into a 1.5 mL EP tube, with 100 μL TCEP solution (100 mg/mL), was followed by shaking for 1 min. Then, 200 μL methanol (containing 500 μg/mL ascorbic acid and 0.1% formic acid) was added, with shaking for another 15 min. After that, the plasma was centrifuged at 13,000 rpm at 4 °C for 15 min, the supernatant stuck to the liner, and the whole thing was placed in a liquid-mass injection bottle for detection.

### 3.3. Standards Preparation

Precisely weigh each standard by gravimetric method, and dissolve them by different solvents. The standards of methionine, serine, betaine and taurine were dissolved in 0.1 M HCl, and the contents were 9.64 mM, 14.44 mM, 2.23 mM, and 7.34 mM, respectively. Other standards, including Hcy, choline, cysteine, glutathione, 5-MTHF, SAH and pyridoxamine, were dissolved in antioxidant solution (100 μg/mL ascorbic acid, 10 mM ammonium acetate, 100 μg/mL DTT) and the contents were 11.22 mM, 1.34 mM, 7.77 mM, 4.74 mM, 1.29 mM, 3.85 mM, 5.22 mM. Take the above standard stock solutions to equal proportions and dilute 10 times with acetonitrile: water (containing 100 μg/mL ascorbic acid, 100 μg/mL DTT and 10 mM ammonium acetate) to obtain standard mixtures.

### 3.4. LC-QTOF-MS Detection Conditions

Chromatographic conditions: Waters XBridge BEH C18 column (2.1*50 mm, 1.7 μm), column temperature: 35 °C, injection volume: 10 μL, flow rate: 0.4 mL/min, equilibration time 1 min, mobile phase A was 5 mM PFHA aqueous solution (containing 0.1% formic acid), mobile phase B was acetonitrile (0.1 formic acid), gradient elution: 0–0.5 min 95% A, 0.5–8 min 95–5%, 8–9 min 5–5%, 9–10 min 5–95%, 10–11 min 95–95%. The total analysis time was 11 min.

Mass spectrometry conditions: Agilent LC-G6500 series quadrupole-time-of-flight mass spectrometer, SECURA125-1CN analytical balance (Sartorius Scientific Instruments Co., Ltd.). MS detection was performed in positive ion (ESI^+^) mode, electrospray voltage was 5.5 kV, sheath gas was 10 units, nebulizer pressure was 35 psi, evaporator temperature was 200 °C, capillary temperature was 380 °C, collision gas (argon gas) was 1.5 mTorr, the scanning time was 0.02 s, the dry N2 gas flow was 14 L/min, and the rest parameters were default parameters.

### 3.5. Data Analysis

Masshunter software was used to extract the metabolite ion chromatograms and the corresponding secondary mass spectrometry information in the samples and standards. The deviation in ppm was set to 20 when extracting the ion chromatogram. The metabolite dataset was log transformed and analyzed by T-test, and *p* < 0.05 indicated a statistical difference. The probabilistic principal component analysis (PPCA) from Metabolomics R package was applied to the dataset after log transformation, with the pareto scale for determining the differential metabolites. The PPCA is an extension of traditional PCA to define an appropriate probabilistic model for PCA. The PPCA can estimate the probability of relevant information while reducing the matrix dimension, so as to obtain more reasonable component information. The Poisson regression analysis was performed by R glmnet package to identify metabolites associated with DM development. Furthermore, the pROC R package was used to perform characteristic curve regression analysis (ROC), where the hazard odds ratio (OR) greater than one indicated a close association with the occurrence of DM. R-based K-means cluster analysis was performed to evaluate the performance of metabolites to distinguish different sample types. The R package ggplot2 visualized all data.

## 4. Conclusions

In conclusion, 13 metabolites related to the Hcy metabolic pathway in DM by LC-QTOF-MS were detected successfully. Four metabolites which highly related to DM, including Hcy, taurine, choline, and methionine, were screened out through different statistical analyses. These metabolites were involved in the methionine cycle, the folate cycle, and the transsulfurization pathway in the Hcy metabolism pathway. Moreover, these four metabolites were closely related to the grading of blood glucose concentration levels. Our data showed the screened four metabolites could serve as the biomarkers for DM diagnosis, as well as the risk factors for the severity of DM.

## Figures and Tables

**Table 1:** Qualitative confirmation of 13 related metabolites in Hcy metabolic pathways.

Metabolites	MZ	RT (min)	MS/MS
Taurine	126.0217	0.67	44.3048, 108.0266
Betaine	118.0941	1.71	58.0649, 59.0727
Glutathione	308.0982	2.39	76.0219, 84.0447
Methionine	150.0582	2.77	56.0497, 104.0533, 133.3201
Choline	104.1069	2.81	59.0013, 61.0124
Serine	105.9539	2.86	61.0107, 88.9056, 70.0637
Hcy	136.0482	3.09	90.0449, 106.944
Dimethylglycine	104.0702	3.89	58.2012
5-MTHF	460.1935	4.01	194.1042, 313.1423, 414.1893
Cystathionine	223.0839	4.26	88.1063, 134.0021
Cysteine	121.9622	4.61	43.3130, 109.0371, 132.004
SAH	385.1318	4.68	88.0221, 134.0269, 250.0746
Pyridoxamine	169.0761	4.94	135.1181, 169.1022

## Data Availability

Not applicable.

## Sample Availability

Samples of the compounds are available from the authors.
